# An automated method for comparing motion artifacts in cine four‐dimensional computed tomography images

**DOI:** 10.1120/jacmp.v13i6.3838

**Published:** 2012-11-08

**Authors:** Guoqiang Cui, Brian Jew, Julian C. Hong, Eric W. Johnston, Billy W. Loo, Peter G Maxim

**Affiliations:** ^1^ Department of Radiation Oncology Stanford University Stanford CA 94305 USA; ^2^ Department of Physics Stanford University Stanford CA 94305 USA

**Keywords:** four‐dimensional computed tomography, motion artifacts, image similarity, normalized correlation coefficient

## Abstract

The aim of this study is to develop an automated method to objectively compare motion artifacts in two four‐dimensional computed tomography (4D CT) image sets, and identify the one that would appear to human observers with fewer or smaller artifacts. Our proposed method is based on the difference of the normalized correlation coefficients between edge slices at couch transitions, which we hypothesize may be a suitable metric to identify motion artifacts. We evaluated our method using ten pairs of 4D CT image sets that showed subtle differences in artifacts between images in a pair, which were identifiable by human observers. One set of 4D CT images was sorted using breathing traces in which our clinically implemented 4D CT sorting software miscalculated the respiratory phase, which expectedly led to artifacts in the images. The other set of images consisted of the same images; however, these were sorted using the same breathing traces but with corrected phases. Next we calculated the normalized correlation coefficients between edge slices at all couch transitions for all respiratory phases in both image sets to evaluate for motion artifacts. For nine image set pairs, our method identified the 4D CT sets sorted using the breathing traces with the corrected respiratory phase to result in images with fewer or smaller artifacts, whereas for one image pair, no difference was noted. Two observers independently assessed the accuracy of our method. Both observers identified 9 image sets that were sorted using the breathing traces with corrected respiratory phase as having fewer or smaller artifacts. In summary, using the 4D CT data of ten pairs of 4D CT image sets, we have demonstrated proof of principle that our method is able to replicate the results of two human observers in identifying the image set with fewer or smaller artifacts.

PACS number: 87.57.cp; 87.57.N‐

## I. INTRODUCTION

Four‐dimensional computed tomography (4D CT) imaging is an important tool in radiation oncology. It enables tighter margins to be used during treatment planning, and enhances accuracy during treatment delivery for patients with tumor motion affected by respiration. The most common method to acquire a 4D CT scan of a patient is to use the CT scanner in cine mode.^(1)^ The time stamps of the reconstructed CT images and the measured respiratory signal of the patient are retrospectively matched. The reconstructed images are sorted either by the phase^(2)^ or by the displacement,^(3)^ which are then stacked to create a three‐dimensional (3D) image of the patient for each image bin. A 4D image set is then reconstructed by viewing the 3D images in sequence for each image bin. The current acquisition and sorting methods led to significant motion artifacts^4^, ^6^ (artifacts in one study measured <4 mm in 90% of scans^(5)^). The occurrence of artifacts is mainly caused by inaccurate determination of the respiratory phase.^(4)^ These artifacts manifest themselves in the CT images as undefined and/or irregular boundaries, consequently degrading image clarity and causing errors in patient contouring and dose calculation.^7^, ^8^

Strategies to reduce motion artifacts and improve 4D CT image clarity have been developed and investigated — for example, the use of audiovisual biofeedback to help improve the patient's respiratory regularity,^9^, ^10^ the application of different algorithms to improve retrospective sorting,^11^, ^13^ and postprocessing of data to improve image reconstruction.^(14)^ However, most of these strategies rely on visual evaluation of the improvements and are, therefore, prone to human subjectivity.

It is the aim of this study to develop an automated method to objectively compare two 4D CT image sets and identify the one with the fewer or smaller artifacts. Ideally, the method should be able to distinguish two or more 4D CT image sets based on the occurrence and severity of their artifacts, and should be able to replicate the findings of human observers. To best of our knowledge this study is the first to provide a tool for the automated and objective evaluation of motion artifacts in 4D CT.

Our method is based on image similarity between edge slices at adjacent couch positions, which is expressed by the normalized correlation coefficient (NCC) between these slices. Of many image similarity metrics, cross‐correlation has been shown to be an effective and useful metric to measure image similarity between respiratory phases of the CT images.^15^, ^17^

## II. MATERIALS AND METHODS

### A. A metric for quantifying motion artifacts based on NCC between two axial CT slices

The NCC $C_{A,B}$ between two axial CT slices *A* and *B* is given by:^(18)^

(1) $$C_{A,B}=\frac{\sum_{u,v}I_{A}(u,v)I_{B}^{*}(u,v)}{\sqrt{\sum_{i,j}I_{A}^{2}(i,j)\cdot \sum_{k,l}I_{B}^{2}(k,l)}}$$

where $I_{A}(u,v)$ and $I_{A}(i,j)$, *and* $I_{B}(u,v)$ and $I_{B}(k,l)$ are the pixel values (representing the Hounsfield Units) of the image slices $I_{A}$ and $I_{B}$, respectively; *u,v,i,j,k,l* ε [0,511] for standard axial CT slices with a field of view of 50 cm; and $C_{A,B}$ can be any value between ‐1 and +1. When two images are identical, $C_{A,B}$ equals +1.

**Figure 1:**
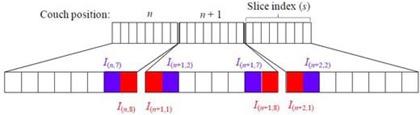
Schematic representation of the couch position (transition) *n*, slice index *s*, edge slices $I_{\left(n,8\right)}$ and $I_{\left(n+1,1\right)}$ at the couch transition *n*, and their neighbors $I_{\left(n,7\right)}$ and $I_{\left(n+1,2\right)}$ in their respective couch positions *n* and n+1.

For a set of retrospectively sorted 4D CT images $I_{\left(n,s\right)}$, n∈[1,N] and s∈[1,S] are the couch position index and the slice index within one couch position, respectively. *N* is the total number of couch positions. The couch transition index is the same as the couch position index, excluding the last couch position. Thus, the total number of couch transitions is N‐1. *S* is the total number of slices in one couch position (i.e., 8 for an 8‐slice scanner). Figure 1 shows the notations for the edge slices $I_{\left(n,8\right)}$ and $I_{\left(n+1,1\right)}$ at the couch transition *n*, and their neighbors $I_{\left(n,7\right)}$ and $I_{\left(n+1,2\right)}$ in their respective couch positions *n* and n+1.

At every couch transition *n*, the NCCs between the edge slices $C_{n}\equiv C_{\left(n,8),(n+1,1\right)}$ and the edge slices and their neighbors in their respective couch positions $C_{n,n}\equiv C_{\left(n,7),(n,8\right)}$ and $C_{n,n+1}\equiv C_{\left(n+1,1),(n+1,2\right)}$ are first calculated using Eq. (1). Based on these three values, we define the following quantity $D_{b,n}$ as a metric of similarity between the edge slices at the couch transition *n* for a given respiratory bin *b*:

(2) $$D_{b,n}={\left[\frac{1}{2}(C_{n,n}+C_{n,n+1})-C_{n}\right]}_{b}$$

**Figure 2:**
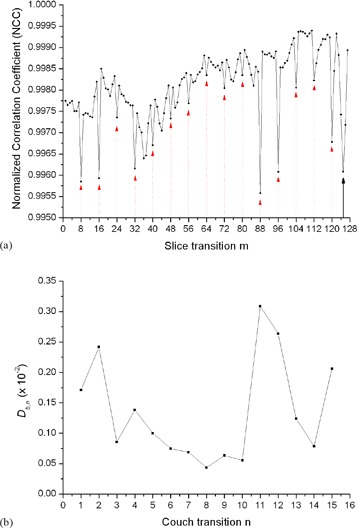
An example (50% phase bin for patient 10) of the NCC $C_{m,m+1}$ between all adjacent slices *m* and m+1 for a scan with 16 couch positions, where m∈[1,127]. The the decrease of the NCC at each couch transition (indicated by the dot arrow lines). Plot (b) of $D_{b,n}$ versus couch transition *n* for this scan.

Since $I_{\left(n,7\right)}$ and $I_{\left(n,8\right)}$ are acquired at the same time within the same breathing cycle, there are no respiratory induced artifacts, and any deviation of $C_{n,n}$ and $C_{n,n+1}$ from +1 is mainly caused by nonrespiratory‐induced anatomical changes (e.g., the transition from the chest wall to the lung), which is possible if a particular couch position contains this transition. In contrast, the edge slices at two adjacent couch positions are acquired in different breathing cycles and can contain respiratory‐induced artifacts, in addition to normal anatomical changes between edge slices. Thus *C* should be no larger than $C_{n,n}$ and $C_{n,n+1}$. (Figure 2a) shows an example (50% phase bin for patient 10) of the NCC $C_{m,m+1}$ between all adjacent slices *m* and m+1 for a scan with 16 couch positions, where m∈[1,127]. The figure shows the decrease of the NCC at each couch transition m=8n, which is smaller than the two neighboring NCCs. However, the decrease of the NCC is not always the largest at the couch transitions, as shown in (Fig. 2a) for m=125. This decrease in the NCC (indicated by the solid arrow line) within the same couch position is caused most likely by abrupt anatomical changes.

The average value of $(C_{n,n}+C_{n,n+1})/2$ represents the baseline similarity in the closest vicinity of the edge slices $I_{\left(n,8\right)}$ and $I_{\left(n+1,1\right)}$. With the use of $D_{b,n}$, we minimize the impact of nonrespiratory‐induced similarity changes in the closest vicinity of couch transitions and emphasize only on the changes (to first order approximation) caused by respiration between edge slices. A plot of $D_{b,n}$ versus couch transition *n* for the same scan of patient 10 is shown in (Fig. 2b). As shown in the figure, the biggest magnitudes of $D_{b,n}$ correspond to couch transitions 11 and 12, which indicates the position of the largest similarity changes and potentially the largest artifacts.

One potential issue with $D_{b,n}$ is the possibility that the similarity between edge slices may not be fully recovered from similarities of surrounding slices, as shown in (Fig. 2a) for m=125. In this case, $D_{b,n}$ would indicate a nonmotion‐related artifact. However, through further processing of the data, which is described further below, this effect can be subtracted out. Here we hypothesize that $D_{b,n}$ is a suitable metric for identifying severity of artifacts and can be used for objectively comparing motion artifacts in cine 4D CT images. We investigated our hypothesis by scoring the artifacts in ten pairs of 4D CT images that showed subtle differences in artifacts between images in a pair.

We have developed a software package with MATLAB (ver. R2011b, The MathWorks, Inc., Natick, MA) that requires already‐sorted 4D CT images as input and then calculates the NCC between edge slices at every couch transition for each respiratory phase bins. The same routine is used for the second set of 4D CT images. Finally, a score is calculated that compares the two 4D CT sets based on the occurrence and severity of artifacts. These steps are fully automated and require only the 4D CT images to be sorted and thus the clinical implementation is readily achievable.

### B. 4d CT image acquisition and sorting

For this study, we identified ten lung cancer patients that were treated at our institution. 4D CT scans for these patients were acquired on a GE Discovery PET/CT Scanner (General Electric Medical Systems, Waukesha, WI) equipped with the real‐time position management (RPM) system (Varian Medical Systems, Palo Alto, CA) for monitoring the patients' breathing, using previously described acquisition techniques.^(19)^ Ten reconstructed phase bins were used, yielding 10 full‐field volumetric image datasets per breathing cycle for each patient. Image processing was performed on an Advantage Workstation 4.2 with Advantage 4D CT software (GE Medical Systems, Waukesha, WI).

**Figure 3:**
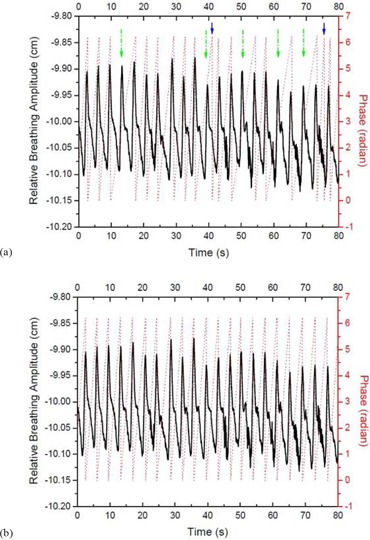
Recorded breathing traces (a) by the RPM software (solid thick curves) with the originally calculated phase values (dotted lines). Five missed peaks and two improperly detected peaks of inhalation are indicated by the long dash‐dot arrows and the short solid arrows, respectively. Recorded breathing traces (b) with recalculated phase values after manually correcting the missed and improperly detected peaks of inhalation.

This patient population was chosen because each patient had a regular breathing pattern that resulted in a regular breathing trace. However, due to miscalculation of the phase by the RPM software, the corresponding 4D CT images showed substantial motion artifacts. We then corrected the respiratory breathing traces using the ‘Phase Recalculation Review’ function in the RPM software, which allowed the manual insertion and/or deletion of inhalation peaks that resulted in different phase values. A representative example of a patients breathing trace with the miscalculated phase (dotted line) due to missed detection of five inhalation peaks (indicated by the long dash‐dot arrows) and two improperly detected inhalation peaks (indicated by the short solid arrows) is shown in (Fig. 3a). After manually adding the missed inhalation peaks and deleting the improperly detected inhalation peaks, the RPM software recalculated the respiratory phase, as shown in (Fig. 3b). We then resorted the original images using this corrected respiratory trace that led to a second set of 4D CT images.

### C. Quantitative evaluation of the 4D CT image sets and generation of the automated scores

To evaluate motion artifacts in the 4D CT images based on the original and corrected RPM traces, we first calculated $D_{b,n}$ at every couch transition *n* for each respiratory phase bin *b* for both 4D CT image sets. This resulted in two matrices $\{D_{b,n}\}O$ and $\{D_{b,n}\}O$, where $\{D_{b,n}\}R$ is generated from the 4D CT images based on the original breathing trace and $\{D_{b,n}\}R$ from the 4D CT images based on the corrected breathing trace with recalculated phase values. Each row of the matrix represents each respiratory phase bin *b* (0%–90%) and the row elements are the $D_{b,n}$ values at every couch transition *n* at that particular phase bin *b*. We then subtracted $\{D_{b,n}\}R$ from $\{D_{b,n}\}O$ to obtain a residual matrix $\{\Delta D_{b,n}\}\equiv {\left\{D_{b,n}\right\}}_{O}‐{\left\{D_{b,n}\right\}}_{R}$. With this, a positive value of the matrix elements Δ$D_{b,n}$ would indicate a higher similarity between the edge slices at couch transition *n* and phase *b* in the 4D CT images that were sorted using the breathing trace with recalculated phase values, compared to the similarity of the edge slices in the 4D CT images based on the original breathing trace. A negative value would indicate the opposite scenario. Consequently, a value of zero would indicate no difference in similarity between the two 4D CT image sets. Next, we summed the row elements for each phase of the {Δ$D_{b,n}$} matrix and assigned it a value of +1 if the sum was positive, ‐1 if the sum was negative, and a value of zero if the sum was zero. The overall score for each patient was generated by averaging the ten values assigned to each phase. With this, the overall score for each patient varied between ‐1 and +1.

### D. Visual comparison of the 4D CT image sets and statistical analysis

To evaluate whether our method has identified the set of 4D CT images that has fewer or smaller artifacts, we developed an image comparison interface that presents images in pairwise fashion to a human observer.^(20)^

Two physicists evaluated image artifacts in the 4D CT sets. For each patient, three coronal and two sagittal cross‐sections were taken from the same locations in two image sets. The two image sets were then displayed side by side, and each observer independently scrolled through the phases of both image sets simultaneously (blinded to the selection method of each image set) and marked the image set that appeared to have fewer or smaller artifacts. A score of +1 was given if the image set sorted using the breathing trace with the recalculated phase values was selected, while a score of ‐1 was given if the image set sorted using the breathing trace with the originally miscalculated phase values was selected. Additionally, the observers had the option to select “neither” if the two image sets were comparable (score was 0). Thus the maximum and minimum score per patient would be +5 and ‐5, respectively. The final score was obtained by averaging the scores of the five image sets. Thus the score per patient would be between ‐1 and +1. A positive score implies that the images sorted using the breathing trace with the recalculated values would result in 4D CT images with fewer or smaller artifacts, while a negative score would imply the opposite. A score of zero implies no difference between the two 4D CT image sets. We furthermore analyzed the interuser variability between the two observers

The statistical analysis of the data was performed using OriginPro 8 SR0 (version 8.0, Northampton, MA). Based on the manual scoring systems described above, an average score for each patient was calculated. A Wilcoxon signed‐rank test was used to determine whether the human average score would yield zero, since the data did not follow a normal distribution. This would indicate that the human observers could not identify any differences between the two 4D CT image sets. A second Wilcoxon signed‐rank test was then used to determine whether the automated score for each patient would yield zero, which would indicate that our method is unable to identify differences between the two 4D CT image sets. Finally, a third Wilcoxon signed‐rank test was used to determine whether there is any statistically significant difference between the scores of the automated method and the average scores determined by the two human observers.

## III. RESULTS

Table 1 summarizes the scoring results of the two observers and our automated method. Listed are the scores from observer #1 and #2, the average score for both observers, and the score determined by our automated method. For nine patients, our method found the 4D CT image sets sorted using the breathing traces with the recalculated phase values to have fewer or smaller artifacts than the 4D CT image sets sorted using the breathing trace with the originally miscalculated phase values. For one patient (patient 5), our method found no difference between the two 4D CT images sets. Overall, our method consistently identified one 4D CT image set to be ‘better’ than the other set and, therefore, the zero hypothesis that our method is unable to distinguish differences between the two 4D CT image sets was rejected (p=0.004). This is in good agreement with the results of the two observers who also consistently identified the 4D CT image set sorted using the breathing trace with the recalculated phase values to result in fewer or smaller artifacts (p=0.004). Both observers found only one case (patient 3) in which the two 4D CT image sets were not different from each other.

**Table 1:** Scoring results of the manual method and automated method for ten patients. Shown are the manual scores by observer 1 and observer 2, the average score of both observers, and the automated score. The scores for both methods range from ‐1 and +1.

*Patient*	*Manual Score*			*Automated Score*
	*Observer 1*	*Observer 2*	*Average*	
1	0.6	0.6	0.6	0.4
2	0.8	1	0.9	1
3	0	0	0	0.4
4	0.6	0.6	0.6	1
5	0.2	0.8	0.5	0
6	0.6	1	0.8	0.4
7	1	1	1	0.6
8	0.2	0.6	0.4	0.4
9	0.8	1	0.9	1
10	0.8	0.8	0.8	0.4

Note: A positive score indicates that the image set sorted using the breathing trace with the recalculated phase values resulted in 4D CT images with fewer or smaller artifacts, whereas a negative score indicate the other image set to have fewer or smaller artifacts. A score of zero indicates no difference between the two image sets.

Next, we have assessed the interuser agreement between the two observers. The results are summarized in Table 2. Shown is the frequency that each observer has chosen a particular 4D CT image set in relation to the other observer. For example, both observers selected the 4D CT image set sorted using the breathing trace with the recalculated phases 29 times to have fewer or smaller artifacts, while they selected the other 4D CT image set to have fewer artifacts once. Furthermore, there was no statistically significant difference between the average score of the two independent observers and the scores determined by our automated method (p=0.43).

**Table 2:** Summary of the image set selection results of two observers. The number represents the frequency both observers found one image set has fewer or smaller artifacts. Of 50 selections, there are complete agreement 40 times (sum of the numbers in the parentheses), partial agreement 9 times (sum of the numbers in the square brackets), and complete disagreement 1 time (sum of the numbers in the curly brackets).

*Observer 1*	*Observer 2*		
	*Image set sorted by recalculated phase*	*Neither*	*Image set sorted by original phase*
Image set sorted by recalculated phase	(29)	[1]	{0}
Neither	[8]	(10)	[0]
Image set sorted by original phase	{1}	[0]	(1)

(Figures 4a)and (4b) show a representative example of the same coronal cross‐section from the two 4D CT image sets (50% phase bin for patient 10), which were sorted using the breathing traces with the originally miscalculated phase values and the recalculated phase values, respectively. (Figure 4a) shows motion artifacts around the dome of the diaphragm, (indicated by the arrows). In contrast, these artifacts around the dome of the diaphragm, as shown in (Fig. 4b), are less prominent.

**Figure 4:**
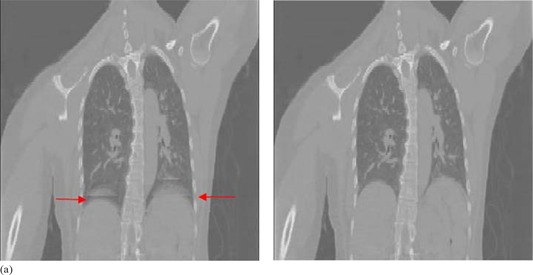
Coronal cross‐sections of the image set of the 50% phase bin for patient 10, sorted using the originally miscalculated phase values (a), and the recalculated phase values (b). Artifacts expressed as overlapping structure in the diaphragm region are indicated by the arrows.

(Figure 5a) shows the difference of the NCC at each adjacent slice between the two 4D CT image sets (50% phase bin for patient 10) as shown in (Figs. 4a)and (4b), respectively. The figure shows little intracouch variation and the largest differences occur at slice transitions 88 and 96, corresponding to couch transitions 11 and 12. Of note is that at m=125, the large decrease in the maximum value of the NCC, as shown in (Fig. 2a), was subtracted out because it occurred intracouch and was static in both 4D CT image sets. (Figure 5b) shows the corresponding Δ$D_{b,n}$ as a function of couch transition for patient 10. According to our metric, the largest motion artifacts occurred at couch transition 11 and 12, which corresponds to the artifacts shown in (Fig. 4a).

**Figure 5:**
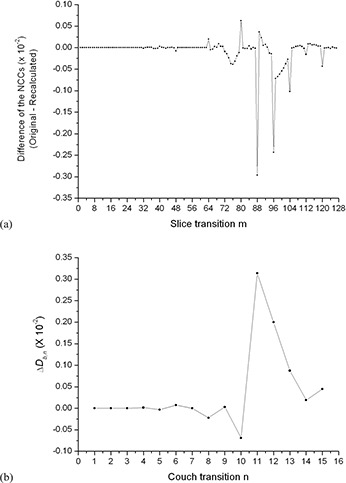
Plot (a) of the difference of the NCC at each adjacent slice between the two 4D CT image sets in (Figs. 4a)and (4b). Plot (b) of Δ$D_{b,n}$ versus couch transition *n* of 50% phase bin image set for patient 10. Motion artifacts occurred at couch transitions 11 and 12.

## IV. DISCUSSION

In this study, we developed an automated method that relies on image similarity between edge slices at couch transitions for the quantification of motion artifacts in cine 4D CT images. Two independent observers validated the proposed method using ten pairs of 4D CT images. Our method was designed to replicate the scoring process of the reviewer who relied on visual inspection of the artifacts. By summing the row elements of the residual matrix {Δ$D_{b,n}$}, we generated a score (‐1, 0, or +1) for each respiratory phase across all couch positions. This resembles the process of visual inspection of a human observer who inspects the entire length of the scanned anatomy and then recognizes whether or not an artifact exists during a particular phase and compares it with the existence and/or the magnitude of the artifact in the other image set. We have chosen this scoring method as visual inspection will tend to identify larger rather than subtle artifacts, and a human observer will therefore identify the image set with the fewer or smaller artifacts to be the ‘better’ one. While visual inspection is inevitably subjective, our method is able to score the image artifacts more objectively, as it will identify any sized artifacts through the calculation of the NCC for edge slices of adjacent couch positions pixel by pixel.

The 4D CT data of ten patients that were analyzed in the context of this study have demonstrated proof of principle that our method is able to differentiate two 4D CT images sets based on their inherent motion artifacts. The fact that our method and the two observers consistently identified the 4D CT image sets that were sorted using the respiratory breathing trace with the recalculated phase values to be the better one, demonstrates that our method is able to perform as well as human observers.

One drawback of our method is the lack of an absolute quantity that describes the goodness of a 4D CT image set. Our method relies on the comparison of two 4D CT images sets to determine the ‘better’ one. In clinical practice, however, it would be desirable to have a metric that will inform the physicians about the quality of the acquired 4D CT images. For example, such information could be used to determine the need of a repeat scan before the end of the patient's simulation and/or determine the appropriate respiratory management technique. The use of $D_{b,n}$ as an overall score for the goodness of a single 4D CT scan is not viable, as is demonstrated in (Fig. 2b). Here the large values of $D_{b,n}$ do not always correspond to the occurrence of a motion artifact. For instance, the relatively large magnitudes of $D_{b,n}$ at couch transitions 1 and 2 are not related to motion artifacts, but are due to abrupt anatomical transitions. However, since they are static and occur in both 4D CT image sets, they can be subtracted out during the scoring process using Δ$D_{b,n}$, as shown in Fig. 5.

Although our method was validated in 4D CT images that were sorted using the same respiratory phase‐based sorting method, it holds potential to be used as a comparison tool for different sorting algorithms, such as respiratory phase‐based and displacement‐based algorithms to determine the one that yields fewer or smaller artifacts.

## V. CONCLUSIONS

An automated method based on image dissimilarity to objectively score motion artifacts in cine 4D CT images retrospectively sorted by respiratory phase‐based sorting was developed. We have demonstrated proof of principle that our method is able to replicate the findings of two human observers in determining the 4D CT images with fewer or smaller artifacts. It holds the potential to be used as a comparison tool for different sorting algorithms and to identify the 4D CT image set with the fewer or smaller artifacts.

## ACKNOWLEDGMENTS

This work was supported by the Mr. and Mrs. Archer Morgan Fellowship in Radiation Oncology at Stanford University.
